# Biomarkers in acute kidney injury

**DOI:** 10.1186/s13613-024-01360-9

**Published:** 2024-09-15

**Authors:** Marlies Ostermann, Matthieu Legrand, Melanie Meersch, Nattachai Srisawat, Alexander Zarbock, John A. Kellum

**Affiliations:** 1 Department of Critical Care, Guy’s & St Thomas’ NHS Foundation Hospital, London, SE1 7EH UK; 2 Department of Anesthesia and Perioperative Care, Division of Critical Care Medicine, University of California San Francisco, San Francisco, USA; 3 Department of Anesthesiology, Intensive Care and Pain Medicine, University Hospital Münster, Münster, Germany; 4 Division of Nephrology, Department of Medicine, Faculty of Medicine, and Center of Excellence in Critical Care Nephrology, Chulalongkorn University, Bangkok, Thailand; 5 Department of Critical Care Medicine, University of Pittsburgh, Pittsburgh, PA USA

## Abstract

Acute kidney injury (AKI) is a multifactorial syndrome with a high risk of short- and long-term complications as well as increased health care costs. The traditional biomarkers of AKI, serum creatinine and urine output, have important limitations. The discovery of new functional and damage/stress biomarkers has enabled a more precise delineation of the aetiology, pathophysiology, site, mechanisms, and severity of injury. This has allowed earlier diagnosis, better prognostication, and the identification of AKI sub-phenotypes. In this review, we summarize the roles and challenges of these new biomarkers in clinical practice and research.

## Introduction

Acute kidney injury (AKI) is a multifactorial syndrome characterised by a rapid (hours to days) deterioration of kidney function and a high risk of short- and long-term complications, effects on quality of life and increased health care costs [1–8]. Despite its frequent occurrence, there are no specific therapies and only limited diagnostic tools used in routine clinical care. Traditionally, AKI is diagnosed by a rise in serum creatinine (sCr) and/or fall in urine output, independent of underlying aetiology, pathophysiology and anatomical site of injury [9]. Both, sCr and urine output are markers of kidney function with important limitations. (Table 1) Further, in most forms of AKI, renal tubular epithelial cells are the primary locus of injury and a reduction in glomerular filtration is a late and insensitive development. In some cases, injury to renal epithelial cells may not even manifest in a functional change and AKI remains undetected (‘subclinical AKI’). Thus, reliance solely on changes in sCr and/or urine output ignores sub-clinical AKI, may delay the recognition and management of AKI and potentially contribute to unfavourable outcomes.

**Table 1:** Limitations of serum creatinine and urine output for assessment of kidney function

Parameter	Limitations
Serum creatinine	• Insensitive to kidney damage especially for health kidneys—nearly 50% of function must be lost before serum creatinine increases • Slow to increase (as long as 24-48 h) following an insult • Affected by exogenous creatine intake • Dependent on muscle mass and metabolism and liver function • May be affected by physiological and analytic variability • Can increase without true change in renal function, for instance in the setting of drug-induced inhibition of tubular secretion (e.g. cimetidine, trimethoprim) or hemoconcentration (e.g. diuresis) • May be altered during pregnancy
Urine output	• Transient oliguria may be physiological • Can be manipulated with diuretics* • Prone to measurement error even with a urinary catheter

^*^Importantly, intact tubular function is necessary for loop diuretics to increase urine output

*AKI* acute kidney injury

Importantly, the serum half-life of creatinine increases from about 4 h to as much as 24–72 h as glomerular filtration rate (GFR) decreases. Thus, the sCr concentration may take 24–36 h to rise after a kidney injury [10]. Further, in patients with sepsis, liver disease, fluid overload and/or muscle wasting, reductions in GFR may not be accurately reflected by sCr and there may be additional delays. A post hoc analysis of the ‘Fluid and Catheter Treatment Trial’ showed that AKI was revealed or classified differently in up to 18% of patients after sCr results were adjusted for net fluid balance and estimated total body water [11]. Affected patients had mortality rates similar to those with AKI that was apparent before adjustment. In addition to the risk of delayed diagnosis, AKI may also be erroneously over-diagnosed, for instance in patients taking drugs that increase creatine availability or compete with tubular secretion, ie, cimetidine or trimethoprim [12].

Lastly, urine production does not correlate with glomerular filtration and may persist until renal function almost ceases. Further, oliguria may be an appropriate physiological response during periods of hypovolaemia, prolonged fasting, after surgery and following stress, pain or trauma [13]. On the other hand, the use of diuretics can increase urine output in patients with AKI without affecting survival [14].

### Adjunctive AKI biomarkers

Additional kidney biomarkers have been discovered and validated in different patient cohorts. They vary in the cells they originate from, their physiological function, their kinetics and distribution. (Table 2) There are 3 broad categories of urinary biomarkers. The first group includes biomarkers which have a low molecular weight and are freely filtered at the glomerulus, e.g. β2-microglobulin, lysozyme, α1-microglobulin, light chains and cystatin C. Under normal circumstances, these molecules are reabsorbed completely by proximal tubular epithelial cells so that they are only detectable in the urine following tubular epithelial cell dysfunction. A second group of biomarkers is upregulated in renal cells in response to kidney injury. These molecules include monomeric neutrophil gelatinase associated lipocalin (NGAL), Dickkopf-3 (DKK3) and kidney injury molecule-1 (KIM-1) which are expressed mainly in distal and proximal tubular epithelial cells [15]. Hepatocyte growth factor (HGF) is a biomarker that is upregulated in interstitial cells in response to kidney injury. A related group of biomarkers are constitutively expressed by tubular epithelial cells and then released into the urine in response to AKI. Examples of this group include the lysosomal enzyme N-acetyl-β-D-glucosaminidase (NAG), the cytosolic protein lactate dehydrogenase or the brush border protein gp130. Cell cycle arrest markers like insulin like growth factor binding protein 7 (IGFBP7) and tissue inhibitor of metalloproteinases 2 (TIMP-2) appear to also be constitutively produced and are released rapidly (i.e. within hours) in response to injurious stimuli causing tubular cell stress [16]. Prolonged and increased expression of TIMP-2 and IGFBP7 may be synonymous with cell damage [17]. C–C motif chemokine ligand 14 (CCL14) is a member of the chemokine family that is implicated in tissue injury and repair processes. It is primarily produced by macrophages and monocytes and is thought to play an important role in recruiting and activating monocytes and other leukocyte subtypes to sites of injury or infection in various organ systems, including the kidney [18, 19]. Lastly, biomarkers may be emitted by inflammatory cells residing or entering the kidneys during AKI. Examples include interleukin-18 (IL-18) which is released by macrophages and neutrophils passing through the kidney and IL-9 which is produced by CD4 + T cells and released into the urine in patients with acute interstitial nephritis [20].

**Table 2:** Description and characteristics of selected AKI biomarkers

AKI biomarker	Biological role	Biological sample	Roles in clinical practice	Populations / settings studied	Limitations / confounders	Regulatory approval
Functional biomarkers						
Cystatin C	13 kDa Cysteine protease inhibitor produced by nucleated human cells; freely filtered	Plasma	Diagnosis of AKI and measurement of severity	ICU Liver transplantation Hospitalized patients	Elevated in CKD Confounded by age, sex, inflammatory state, diabetes, low albumin, glucocorticosteroids	FDA approved and CE marked for GFR estimation in clinical practice
Proenkephalin A	Endogenous polypeptide hormone in adrenal medulla, immune system and renal tissue; freely filtered and measured in plasma	Plasma	Diagnosis and assessment of AKI severityI and renal recovery	Hospitalized patients	Elevated in CKD may be less sensitive than creatinine or cystatin C	CE marked for clinical use
Stress biomarkers						
Dickkopf-3 (DKK3)	38 kDa Renal tubular cell–derived glycoprotein; secreted into urine under tubular stress conditions	Urine	Risk assessment and prediction of AKI	Cardiac surgery	Elevated in CKD	
Tissue inhibitor of metalloproteinases-2 (TIMP-2); Insulin-like growth factor binding protein 7 (IGFB7)	Proteins released by tubular epithelial cells (TIMP-2 distal, IGFBP7 proximal) that can induce cell cycle arrest	Urine	Prediction and diagnosis of AKI and assessment of severity	ICU		US FDA and EC marked for predicting stage 2 or 3 AKI in adults
Damage biomarkers						
Alanine aminopeptidase (AAP); γ-glutamyl transpeptidase (γ-GT); Alkaline phosphatase (ALP)	Enzymes located in several organs, including the brush border of proximal tubular cells	Urine	Diagnosis and severity of AKI	ICU	Elevated in UTI, cardiovascular disease, stroke	
C–C motif chemokine ligand 14 (CCL 14)	Pro-inflammatory chemokine implicated in tissue injury and repair processes; primarily produced by macrophages and monocytes; has a role in activating monocytes, recruiting immune cells to sites of injury or infection and in regulating inflammation in various organ systems	Urine	Persistence of severe AKI	ICU		
Chitinase 3-like protein 1	39 kDa Intracellular protein of glycoside hydrolase family; expressed by endothelial cells, macrophages & neutrophils and released into the urine and plasma	Urine Plasma	Diagnosis of AKI	ICU	Limited performance in real-world settings as a single biomarker	
Hepatocyte growth factor (HGF)	Antifibrotic cytokine produced by mesenchymal cells; involved in tubular cell regeneration after AKI and measured in plasma	Plasma	Severity of AKI and renal recovery	Hospitalized	Limited performance	
Hepcidin	2.78 kDa Peptide hormone predominantly produced in hepatocytes; freely filtered into urine and plasma	Urine Plasma	Diagnosis of AKI and assessment of severity	ICU	Decreased in anemia and increased in inflammatory state	
Interleukin-18 (IL-18)	18 kDa Pro-inflammatory cytokine; released into urine following tubular cell damage	Urine	Prediction and diagnosis of AKI	Hospitalized patients ED	Elevated in inflammatory states	
Interleukin-9 (IL‑9)	30-40 kDa Pleiotropic cytokine secreted by CD4 + helper cells	Urine	Differential diagnosis of AKI	Acute interstitial nephritis		
Kidney Injury Molecule–1 (KIM-1)	Transmembrane glycoprotein produced by proximal tubular cell; released into urine after tubular cell damage	Urine	Prediction and diagnosis of AKI and assessment of severity	Hospitalized patients ED	May take 24 h to more to peak after injury	US FDA approval and EC marked for pre-clinical drug development
Liver-type fatty acid-binding protein (L-FABP)	14 kDa Intracellular lipid chaperone; freely filtered and reabsorbed in proximal tubule; excreted into urine after tubular cell damage and measured in the urine and plasma	Urine Plasma	Diagnosis of AKI	ICU ED	Associated with anemia in non-diabetic patients	Japan Ministry of Health, Labour, and Welfare approval for clinical use
N-acetyl-β-D-glucosaminidase (NAG)	> 130 kDa Lysosomal enzyme; released into urine after tubular damage	Urine	Diagnosis of AKI	Hospitalized patients	Elevated in diabetes and albuminuria	
Neutrophil gelatinase-associated lipocalin (NGAL)	At least 3 different types measured in the urine and plasma: • i) Monomeric 25 kDa glycoprotein produced by neutrophils and epithelial tissues, including tubular cells ii) Homodimeric 45 kDa protein produced by neutrophils iii) Heterodimeric 135 kDa protein produced by tubular cells	Urine Plasma	Diagnosis of AKI and measurement of severity	Hospitalized patients ED	Elevated in sepsis, UTI, CKD Measurement of general inflammation	US FDA approved for children; CE marked

*AKI* acute kidney injury, *CKD* chronic kidney disease, *ED* emergency department, *ICU* intensive care unit, *UTI* urinary tract infection, *TNFα* tumor necrosis factor α, *GFR* glomerular filtration rate, *FDA* Food and Drug Administration, *EC* European Conformity, *CE* Conformité Européene

Another method of categorising renal biomarkers is to stratify them based on their characteristics. Thus, some biomarkers primarily reflect glomerular filtration (i.e. serum cystatin c, proenkephalin A), glomerular integrity (i.e. albuminuria and proteinuria), tubular stress (i.e. IGFBP7 and TIMP-2), tubular damage [i.e. NGAL, KIM-1, NAG, Liver fatty acid-binding protein (L-FABP)], intra-renal inflammation (i.e. IL-18, IL-9) or repair mechanisms (CCL14).

### Role of AKI biomarkers in clinical practice

**Fig. 1:**
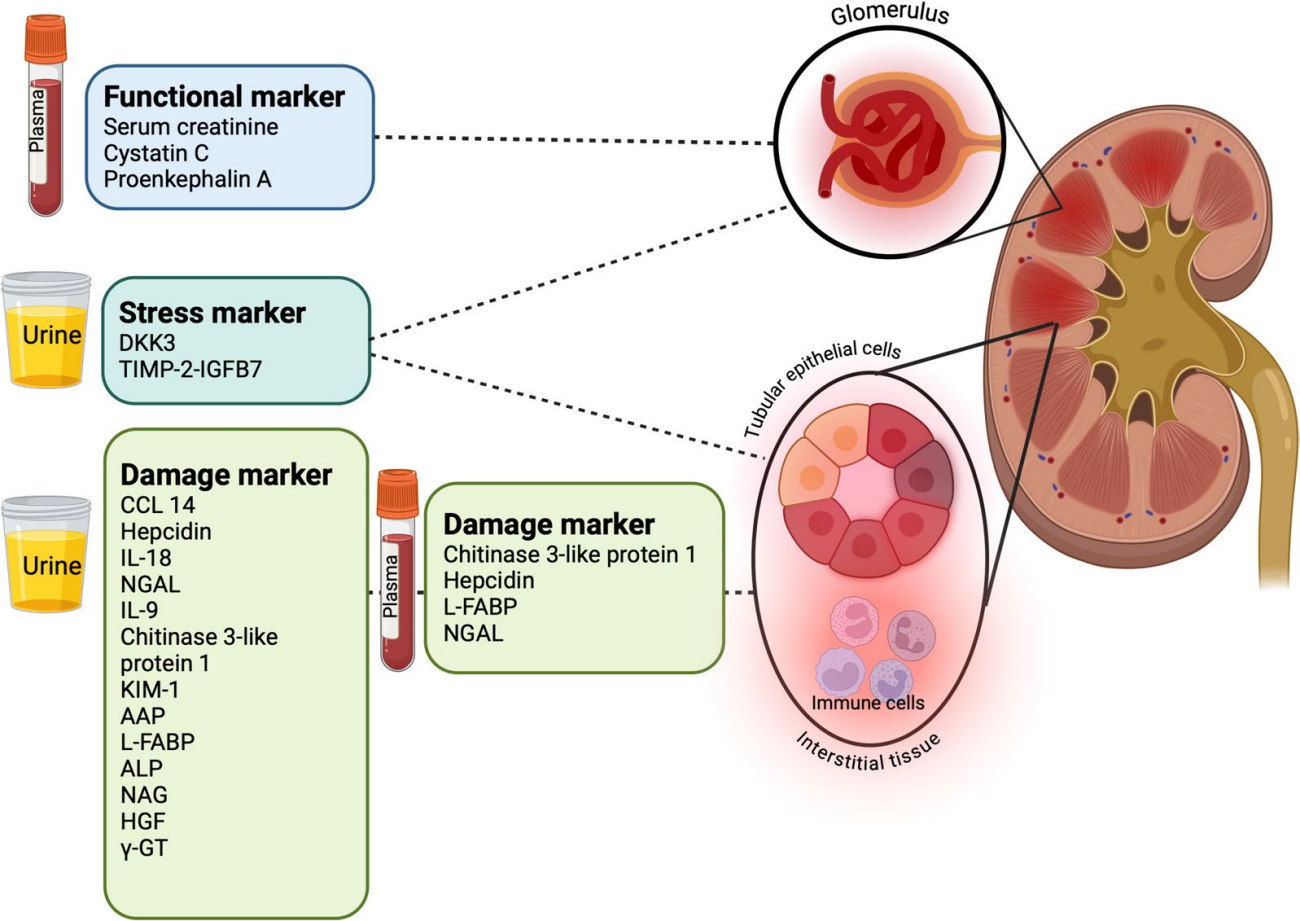
Different types of AKI biomarkers. *AAP* Alanine aminopeptidase, *ALP* Alkaline phosphatase, *CCL14* C–C motif chemokine ligand 14, *DKK3* Dickkopf-3, *γ-GT* γ-glutamyl transpeptidase, *HGF* Hepatocyte growth factor, *IGFBP7* Insulin like growth factor binding protein 7, *IL-9* Interleukin-9, *IL-18* Interleukin-18, *KIM-1* Kidney injury molecule-1, *L-FABP* Liver fatty acid-binding protein, *NAG* N-acetyl-β-D-glucosaminidase, *NGAL* Neutrophil gelatinase associated lipocalin, *TIMP-2* Tissue inhibitor of metalloproteinases-2

The discovery of these new functional and damage/stress biomarkers has enabled a more precise delineation of the aetiology, pathophysiology, site, mechanisms, and severity of injury, earlier diagnosis and better prognostication, and the identification of AKI sub-phenotypes [21–25]. (Table 2, Fig. 1) However, it is important to acknowledge that biomarkers have specific characteristics and kinetic profiles. Since no biomarker can fulfil all these tasks, they need to be matched with their respective purpose and be measured at appropriate time points.

- (i) Early diagnosis of AKI

Several biomarkers may indicate the early onset of AKI and enable the identification of patients with evidence of kidney injury without a change in sCr, ie. “sub-clinical AKI” [21, 22, 26–28]. A study in 178 children showed that those who had elevated urine NGAL (uNGAL) concentrations without a rise in sCr had an almost fourfold increased risk of all-stage AKI on day 3 compared to children without an uNGAL and sCr rise [29]. Two meta-analyses including 19 studies with 2,538 patients and 16 studies with 2,906 patients, respectively, found NGAL to be an useful early indicator of AKI, both overall and across a range of clinical settings [30, 31]. The Sapphire study showed that urinary [TIMP-2] × [IGFBP7] concentration was superior to all other biomarkers in critically ill patients for predicting the development of moderate / severe AKI in the following 12 h with an area under the receiver operating curve (AUC) of 0.80 [32]. The risk for major adverse kidney events (MAKE) including death, dialysis or persistent renal dysfunction within 30 days was significantly increased if urinary [TIMP-2] x [IGFBP7] > 0.3 (ng/ml)^2^/1,000 and doubled when values were > 2.0 (ng/ml)^2^/1,000. A follow-up analysis showed that combining [TIMP-2] x [IGFBP7] with serum creatinine significantly (p = 0.02) increased the AUC from 0.80 [95% confidence interval CI 0.76–0.84] to 0.85 (95% CI 0.81–0.89) [33]. A meta-analysis of 20 studies including 3625 patients concluded that urinary [TIMP-2] × [IGFBP7] was a reliable effective predictive test for all cause-AKI with an AUC of 0.81 [34].

The role of proenkephalin A as a filtration marker was confirmed in a meta-analysis of 11 observational studies with 3969 patients showing a pooled AUC of 0.77 (95% CI 0.73–0.81) [35]. However, it was acknowledged that the small sample sizes in the majority of the included studies could potentially lead to an overestimation of effects.

- (ii) Determining AKI aetiology

AKI is not a single disease but rather a multi-factorial syndrome characterized by a wide spectrum of pathophysiological processes. Some biomarkers have proven to be useful in identifying the main aetiology and/or excluding potential causes [33, 36, 37]. For instance, in patients with acute decompensated heart failure treated with diuretics, a rise in sCr is often observed, sometimes prompting clinicians to stop diuretics. However, there is evidence that further decongestion in this context is associated with reduced mortality, despite the apparent functional AKI [38]. In this context, observational studies have revealed that the sCr rise was often not associated with an increase in urinary NAG, KIM-1 or NGAL [36]. Thus, determining whether an increase in sCr represents kidney damage or simply reflects diuretic-induced free water removal is useful. Similarly, in patients with cirrhosis, differentiating between hepato-renal syndrome (HRS)-AKI and other types of AKI is challenging. A recent expert consensus meeting by the Acute Disease Quality Initiative (ADQI) and the International Club of Ascites (ICA) concluded that the combined use of functional (*e.g.*, sCr, Cystatin C) and damage (*e.g.*, albuminuria, uNGAL) biomarkers enabled more accurate differential diagnosis of the aetiology and mechanisms of AKI in patients with cirrhosis and potentially enabled the identification of AKI sub-phenotypes suitable for specific therapeutic interventions [39, 40]. For instance, in 162 patients with cirrhosis and AKI, the response rate to terlipressin was 70% in patients with uNGAL < 220 μg/g of creatinine compared to only 33% in those with uNGAL > 220 μg/g of creatinine [41]. Subanalyses of the Sapphire study showed the kinetics of cell cycle arrest biomarkers after exposure to nephrotoxic insults and allowed the identification of patients with nephrotoxin induced AKI [37, 42].

Urinary IL‑9 has emerged as a biomarker suggestive of the diagnosis of acute interstitial nephritis (AIN) versus acute tubular injury, glomerular diseases, and diabetic kidney disease [20, 43]. Moreover, among patients with AIN, those with high urinary IL-9 levels benefited most from therapy with corticosteroids.

- (iii) Prediction of persistent AKI

The distinction between transient (< 48 h) and persistent (> 48 h) AKI is clinically important as a persistent AKI is associated with worse outcomes [44, 45]. Further, the duration of AKI impacts clinical management. For instance, the use of renal replacement therapy (RRT) is typically reserved for patients with persistent AKI. In 331 critically ill patients with AKI stage 2 or 3, the urinary biomarker CCL14 was found to identify those with a high risk of delayed recovery [19]. A meta-analysis of 6 studies including 952 patients with AKI concluded that urinary CCL14 was an effective marker for predicting persistent AKI with a pooled diagnostic accuracy of 0.84 [46]. This offers opportunities to improve patient outcomes by enabling individualised and targeted interventions appropriately [47].

Similarly, a study evaluating the role of proenkephalin A in sepsis / septic shock showed that the proenkephalin levels within 24 h of ICU admission were significantly higher in those patients who subsequently developed MAKE or persistent or worsening AKI [48]. Finally, in 733 patients undergoing cardiac surgery, preoperative ratio of urinary DKK3 to creatinine concentrations greater than 471 pg/mg was associated with a significantly higher risk of persistent kidney dysfunction (OR 6.67; 95% CI 1.67–26.61, p = 0.0072) and dialysis dependency (OR 13.57; 95% CI 1.50–122.77, p = 0.020) after 90 days compared with a ratio of 471 pg/mg or less [49].

- (iv) Prediction of RRT

Receipt of RRT is a common endpoint of biomarker analysis. Several biomarkers, including NGAL, IL-18, cystatin C, cell cycle arrest markers and CCL14 associate with RRT initiation. For instance, analysis of data from the Protocolized Care for Early Septic Shock (ProCESS) trial showed that patients who still had an urinary [TIMP-2] x [IGFBP7] concentration > 0.3 (ng/ml)^2^/1,000 after receiving fluid resuscitation were at higher risk for a composite endpoint of progression to AKI stage 2/3, receipt of RRT or mortality [50]. A previous systematic review and meta-analysis including 63 studies comprising 15,928 critically ill patients published until September 2017 concluded that several biomarkers showed reasonable prediction of RRT use for critically ill patients with AKI but the strength of evidence precluded their routine use to guide decision-making on when to initiate RRT [51]. Studies using CCL14 were not included.

It is important to point out that timing of RRT remains heterogenous in clinical practice and advice regarding indications and timing has changed over time [52–54]. Further, most studies evaluated biomarker performance by their ability to predict “receipt of RRT” rather than “meeting specific indications for RRT”, independent of whether RRT was initiated or not.

Finally, there may be a role for biomarkers in combination with other tests. For instance, an observational study in 208 critically ill patients showed that the combination of the furosemide stress test with urinary CCL14 results had better predictive ability than the CCL14 result in isolation [55]. The combination of a negative furosemide stress test and high urinary CCL14 levels had a predictive value for the development of an indication for RRT with an AUC of 0.87 (95% CI 0.82–0.92).

- (v) Prediction of long-term outcomes after AKI

Patients surviving an episode of AKI are at high risk of chronic kidney disease (CKD), cardiovascular morbidity and reduced long-term survival [3, 56, 57]. Serum creatinine has a limited role in indicating kidney health in the acute setting, in particular in patients with muscle wasting and persistent morbidities.

In a prospective study of 164 critically ill patients with AKI, urinary CCL14, [TIMP-2] x [IGFBP7] and NGAL, sampled at the time of AKI onset, were studied to assess their predictive ability for renal non-recovery within 7 days [58]. While NGAL was not predictive, CCL14 (assayed with an ELISA rather than the clinical test) had fair prediction ability (AUC 0.71 [95% CI 0.63–0.77]) and [TIMP-2] x [IGFBP7] had the best predictive ability for renal non-recovery (AUC 0.78 [95% CI 0.71–0.84]). A secondary analysis of the Sapphire study showed a higher risk of death and RRT over 9 months of follow-up in critically ill patients with AKI and [TIMP-2] x [IGFBP7] concentrations > 0.3 (ng/ml)^2^/1000 at the time of study enrolment, compared to those with levels ≤ 0.3 (HR 1.44 [95% CI 1.00—2.06]) [59].

Similarly, an elevated Proenkephalin A level at ICU admission in patients not meeting the sCr and urine output criteria for AKI was associated with an increased risk of death close to patients with AKI [60]. A secondary analysis of a multicentre, prospective cohort study of critically ill patients explored the association between renal biomarkers at time of ICU discharge and 1-year mortality and showed that elevated plasma cystatin C, plasma NGAL, uNGAL, and Proenkephalin A levels at ICU discharge were associated with a higher risk of 1-year all-cause mortality, including in patients with low sCr results at discharge [61]. Coca et al. studied the association between 5 urinary kidney injury biomarkers including NGAL, IL-18, KIM-1, Liver-type Fatty Acid Binding Protein (L-FABP), and albumin and all-cause mortality in a multicenter, prospective 3 years follow-up study in six clinical centers in the United States and Canada including 1199 adults who underwent cardiac surgery [62]. The highest tertile of biomarkers was associated with a 2 to 3.2 fold increased risk of mortality when compared with the lowest tertile. Together, these findings suggest that several biomarkers have potential to identify high risk patients, though they have not yet been studied to guide follow-up care.

- (vi) Identification of AKI sub-phenotypes

It is now clear that the syndrome (i.e., phenotype) of AKI is comprised of numerous sub-phenotypes, which can be identified and discriminated through shared features such as etiology/cause, risk factors, diagnostic features and prognosis [63–65]. Importantly, identified sub-phenotypes may behave differently in response to selected interventions. Existing and emerging biomarkers are likely to have a prominent role in discriminating sub-phenotypes of AKI directly, if they are specific for a particular sub-phenotype, or indirectly, if the absence of a detectable biomarker excludes that sub-phenotype [43, 66].

Progress in precision medicine has been furthered through advances in AKI sub-phenotyping, facilitated by novel biomarkers of AKI combined with leveraging electronic health records and advanced analytical methods. For instance, in 2 cohorts of septic patients, latent class analysis (LCA) identified 2 distinct AKI sub-phenotypes defined by different levels of biomarkers [66]. One type had a greater risk of renal non-recovery and 28-day mortality compared to the other. Using data from the VASST trial (vasopressin versus norepinephrine infusion in patients with septic shock), the authors observed that these sub-phenotypes had heterogeneity of treatment effect for the early addition of vasopressin.

Expanding the diagnostic criteria of AKI by integrating biomarkers of kidney stress/damage will not only embrace the concept of sub-clinical AKI but can also enable further differentiation of sub-phenotypes of AKI [67].

### Biomarker-guided management

Several studies have shown benefit associated with the use of functional and damage/stress biomarkers, in particular after surgery and in the context of nephrotoxin exposure [22]. The ‘Prevention of cardiac surgery associated AKI by implementing the KDIGO guidelines in high-risk patients identified by biomarkers’ (PrevAKI) trial was the first which used biomarkers to identify high risk patients [26]. In this single-center randomized controlled trial, patients were analyzed for risk of AKI by measuring urinary [TIMP-2] x [IGFBP7] 4 h after cardiopulmonary bypass surgery. Patients with an urinary [TIMP-2] x [IGFBP7] concentration ≥ 0.3 (ng/ml)^2^/1000 were randomized to receive a care bundle which was based on the recommendations by the Kidney Diseases Improving Global Outcomes (KDIGO) expert panel with focus on haemodynamic and fluid optimisation and avoidance of nephrotoxic exposures. The proportion of patients with any stage AKI was significantly lower in the intervention group compared to the control arm [55.1 versus 71.7%; adjusted risk ratio (ARR) 16.6% (95 CI 5.5–27.9%]); p = 0.004). Rates of moderate to severe AKI were also significantly reduced by the intervention compared to controls (29.7% versus 44.9%; p = 0.009). There were no differences in rates of RRT, mortality or persistent renal dysfunction at 30, 60 or 90 days. A follow-up study in 12 international sites confirmed these findings [28]. The occurrence of moderate and severe AKI was significantly lower in the intervention group compared to the control group [14.0% vs 23.9%; ARR 10.0% (95% CI 0.9–19.1); p = 0.034]. Again, there were no significant differences in other specified secondary outcomes.

In the ‘Biomarker-guided intervention to prevent AKI after major surgery (BigPAK)’ trial, a similar care bundle including early optimization of fluids and maintenance of perfusion pressure, was applied to patients after non-cardiac major surgery who were testing positive for the urinary [TIMP-2] × [IGFBP7] biomarker [27]. Although there was no statistically significant difference in total AKI incidence between the groups (31.7% in the intervention group versus 47.5% in the standard care group, p = 0.076), the rates of AKI stage II/III were reduced in the intervention group (6.7% versus 19.7%, p = 0.04), as were length of stay in ICU (median difference 1 day, p = 0.03) and in hospital (median difference 5 days, p = 0.04).

The role of measuring urinary [TIMP-2] × [IGFBP7] in high risk patients attending the emergency department was evaluated in a single center study [68]. One hundred patients with a urinary [TIMP-2] × [IGFBP7] concentration > 0.3 were randomized to immediate one-time nephrological consultation and implementation of the KDIGO AKI recommendations versus usual care. There was no significant difference in the primary outcome (incidence of moderate to severe AKI within the first day after admission) but patients in the intervention arm had significantly lower sCr results on day 2, lower maximum sCr results and a higher urine output on day 3. Adequately powered trials are needed to confirm these results [69].

Biomarker guided management in sepsis was evaluated in the LAPIS trial [70]. This study compared a three-level kidney-sparing sepsis bundle based on the KDIGO recommendations and guided by risk stratification using serial measurement of urinary [TIMP-2] x [IGFBP7] in patients with sepsis. The study was terminated prematurely and concluded that this strategy was feasible and safe.

Nephrotoxins contribute to approximately 30% of AKI cases in critically ill patients. The measurement of biomarkers has been shown to support drug stewardship, informing medication prescription, drug dosing as well as the application of supportive measures. In a real-world evaluation, the use of urinary [TIMP-2] x [IGFBP7] as an AKI risk screening tool resulted in differential application of various components of the AKI management bundle to those with a positive test result [71]. 51% of patients had at least one medication change in response to a [TIMP-2] x [IGFBP7] result. NGAL has been evaluated for cisplatin-associated AKI and demonstrated that NGAL facilitated the recognition of AKI 4.5 days sooner than sCr for cisplatin associated AKI [72]. In patients with amphotericin-induced AKI, NGAL detected the event about 3 days sooner than sCr [73].

Biomarkers can also be used to classify potentially nephrotoxic drugs and determine their direct effect on the kidneys. [74] Further, panels of tubular damage markers have been approved for assessment of tubular injury in non-clinical and clinical drug studies to help guide safety assessments of new potential therapies [75].

### Biomarker adoption into clinical practice

The increasing ability to measure new AKI biomarkers and the observation that they likely identify an earlier phase of AKI and allow sub-phenotyping has opened the door to personalised medicine for AKI [76]. In the opinion of many experts, this approach is ready for daily use in conjunction with traditional tests [47, 77, 78]. However, the adoption into routine clinical practice has been slow. The reasons vary between institutions but include several factors. First, there is heterogeneity in the performance of various biomarkers in different clinical studies, depending on the patient cohort, timing of measurement, chosen cut-off values, laboratory technique (including use of research assays rather than approved clinical tests) and confounding factors. Second, specific cut-offs have not been determined for all biomarkers. Third, standardized assays and point-of-care testing devices are not available for all biomarkers, and not all biomarker tests are approved by regulatory bodies for clinical use. Fourth, financial costs are often mentioned as barriers. However, a recent cost-utility analysis of biomarkers predicting persistent AKI concluded that biomarker-directed care led to lower total costs and more quality adjusted life years and was cost-effective as the $50,000/QALY threshold [79].

The 23rd ADQI panel concluded that a combination of damage and functional biomarkers, along with clinical information, improved the diagnostic accuracy of AKI, allowed the recognition of different pathophysiological processes, could discriminate AKI aetiology and served to assess AKI severity [67]. Further, the expert panel proposed that clinical information enriched by damage and functional biomarkers could lead to more sensitive AKI definitions and assist the prevention and management of AKI. However, the expert panel was not able to issue specific recommendations for all potential clinical scenarios [67].

## Conclusions

Kidney biomarkers have a role in diagnosing AKI earlier, differentiating between different sub-phenotypes of AKI, identifying high-risk patients and informing clinical management in specific clinical scenarios. The role of individual biomarkers depends on their specific characteristics. To achieve personalised AKI management, integration of appropriate AKI biomarkers into routine clinical practice is essential.

## Data Availability

Not applicable.

## Abbreviations

AAP: Alanine aminopeptidase
ADQI: Acute disease quality initiative acute disease quality initiative (ADQI)
AIN: Acute interstitial nephritis
AKI: Acute kidney injury
ALP: Alkaline phosphatase
ARR: Adjusted risk ratio
AUC: Area under the receiver operating curve
BigPAK: Biomarker-guided intervention to prevent AKI after major surgery
CCL14: C–C motif chemokine ligand 14
CI: Confidence interval
CKD: Chronic kidney disease
DKK3: Dickkopf-3
ED: Emergency department
GFR: Glomerular filtration rate
γ-GT: γ-Glutamyl transpeptidase
HGF: Hepatocyte growth factor
HRS: Hepato-renal syndrome
ICA: International club of ascites
ICU: Intensive care unit
IGFBP7: Insulin like growth factor binding protein 7
IQR: Interquartile range
IL-9: Interleukin-9
IL-18: Interleukin-18
KDIGO: Kidney diseases improving global outcomes
KIM-1: Kidney injury molecule-1
LCA: Latent class analysis
L-FABP: Liver fatty acid-binding protein
MAKE: Major adverse kidney events
NAG: N-acetyl-β-D-glucosaminidase
NGAL: Neutrophil gelatinase associated lipocalin
OR: Odds ratio
PrevAKI: Prevention of cardiac surgery associated AKI by implementing the KDIGO guidelines in high-risk patients identified by biomarkers
ProCESS: Protocolized care for early septic shock
RRT: Renal replacement therapy
SCr: Serum creatinine
TIMP-2: Tissue inhibitor metalloproteinase 2
TNFα: Tumor necrosis factor α
uNGAL: Urine NGAL
UTI: Urinary tract infection
VASST: Vasopressin versus norepinephrine infusion in septic shock
